# Development and validation of a predictive model to guide the use of plerixafor in pediatric population

**DOI:** 10.1038/s41409-022-01831-2

**Published:** 2022-09-26

**Authors:** Bernard Sebastien, Peter Cheverton, Catherine Magnin, Jihane Aouni, Remi Castan

**Affiliations:** 1 Sanofi, Chilly-Mazarin, France; 2 Cheverton Oncology Consultants Ltd, Bury St Edmunds, UK; 3 Sanofi, Gentilly, France; 4 Ividata Group, Levallois-Perret, France

**Keywords:** Stem-cell research, Haematopoietic stem cells

## Abstract

Plerixafor, a CXCR4 receptor antagonist, reduces the binding and chemotaxis of hematopoietic stem cells to the bone marrow stroma, resulting in predictable peak of cluster of differentiation 34^+^ (CD34^+^) cells in the peripheral blood (PB) approximately 10 h after its administration. We developed a model that could predict the CD34^+^ harvest volume on the first day of apheresis (AP-CD34^+^) based on PB-CD34^+^ counts immediately prior to commencing apheresis in pediatric population. In all, data from 45 pediatric patients from the MOZAIC study who received either granulocyte colony-stimulating factor (G-CSF) alone or G-CSF plus plerixafor were included. The modeling of the data exhibited a strong and highly predictive linear relationship between the counts of PB-CD34^+^ cells on the first day of apheresis and AP-CD34^+^ cells collected on the same day. It is predicted that there are approximately 13 new collected CD34^+^ cells for 100 new circulating CD34^+^ cells before apheresis. Our predictive algorithm can be used to quantify the minimal count of PB-CD34^+^ cells that enables to collect at least 2 × 10^6^ or 5 × 10^6^ AP-CD34^+^ cells/kg with sufficient assurance (probability = 0.90) and can guide the use of plerixafor in patients at higher perceived risk for mobilization failure. Trial registration of MOZAIC study: ClinicalTrials.gov, NCT01288573; EudraCT, 2010-019340-40.

## Introduction

Autologous stem cell transplant is an integral life-saving treatment option for several pediatric cancers including high-risk neuroblastoma, central nervous system tumors, certain lymphomas, and for some patients with Ewing’s sarcoma [1–5]. Each diagnosis requires an array of treatment modalities including chemotherapy, surgery, and/or hematopoietic stem cell (HSC) transplantation depending upon the disease pathology, genetic abnormalities, and other disease features.

Mobilized peripheral blood (PB) stem cells collected by apheresis are the preferred source of HSCs in autologous transplantation setting. Successful mobilization of PB stem cells and adequate stem cell collection are of critical importance, as the dose of PB-cluster of differentiation 34^+^ (PB-CD34^+^) cells can be used to assess the PB progenitor cell graft quality and predict hematopoietic recovery after engraftment. Doses of 2 × 10^6^ to 5 × 10^6^ CD34^+^ cells/kg body weight are associated with more rapid engraftment and a lower probability of graft failure and are inversely related to resource utilization for patients up to 100 days after initial therapy [5–7].

Mobilization is generally performed with granulocyte colony-stimulating factor (G-CSF) with or without chemotherapy in pediatric patients. However, these mobilization regimens have 8–27% failure rate among healthy donors and patients [8–10]. The rapid CXCR4/CXCL12 chemotaxis pathway blockage by plerixafor (Mozobil^®^, Sanofi, Cambridge, MA, USA), a CXCR4 antagonist, and synergy with G-CSF and chemotherapy have been shown to be effective in adults [11].

Plerixafor reduces binding and chemotaxis of HSCs to bone marrow stroma, resulting in predictable peak of PB-CD34^+^ cells approximately 10 h after its administration [12–14]. Plerixafor has been shown to successfully mobilize CD34^+^ cells when used concomitantly with granulocyte colony-stimulating factor (G-CSF) in adults failing or likely to fail standard mobilization with G-CSF or G-CSF combined with chemotherapy. A reduction in the number of apheresis days and an increase in CD34^+^ cell mobilization yield was observed in two phase-3 studies in adults with multiple myeloma (MM) [15] and non-Hodgkin’s lymphoma (NHL) [16] when plerixafor was administered on and beyond the fourth day of G-CSF administration in comparison to G-CSF alone.

Plerixafor in combination with G-CSF was approved in the USA to enhance mobilization of HSCs to PB for collection and subsequent autologous transplantation in adults with NHL or MM [17]. In the European Union, plerixafor is indicated in combination with G-CSF for use in adults with lymphoma and MM who are proven to be poor mobilizers [18]. Additionally, it is indicated in combination with G-CSF to enhance mobilization of HSCs to PB for collection and subsequent autologous transplantation in children (aged 1 year to <18 years) with lymphoma or solid malignant tumors, either pre-emptively, when circulating stem cell count on the predicted day of collection after adequate mobilization with G-CSF (with or without chemotherapy) is considered to be insufficient with regards to desired HSCs yield, or in pediatric patients who previously failed to collect sufficient HSCs [18].

While the efficacy and safety of plerixafor is well established in adults, limited data are available for its use in children. In the pediatric setting, the efficacy and safety of plerixafor were evaluated in the MOZAIC trial, an open-label Phase-2 controlled study [4, 19].

Mobilization algorithms can guide the use of plerixafor in patients at a higher perceived risk for mobilization failure along with better utilization of available resources.

The objective of the extrapolation study was to demonstrate that PB-CD34^+^ counts immediately prior to commencing apheresis were indicative of the CD34^+^ cells harvest collected after apheresis in the pediatric population and to quantify the relationship between the two.

## Material and methods

The MOZAIC study (NCT01288573; EudraCT2010-019340-40) [4, 19] was a Phase 1/2 combined dose-ranging study (Stage-1) followed by a randomized, open-label study (Stage-2). In Stage-2, efficacy and safety of plerixafor (240 µg/kg) + G-CSF was assessed for mobilization of HSCs into PB, and subsequent collection by apheresis, versus G-CSF regimen alone (2:1) in 45 pediatric patients with solid malignant tumors or lymphoma eligible for autologous transplants, aged 1 to <18 years [4, 19]. More patients in the plerixafor arm (24/30, 80%) met the primary endpoint of successful mobilization (doubling of peripheral blood CD34+ cell count in the 24 h prior to first apheresis) than in the G-CSF arm (4/14, 28.6%, *p* = 0.0019).

This predictive model for CD34^+^ counts considered pediatric patients from Stage-2 of the MOZAIC study. The data included was limited to the first day of apheresis as CD34^+^ cells collection period in the MOZAIC study was limited to a single apheresis day in > 84% (38/45) of patients.

### Model development

**Fig. 1:**
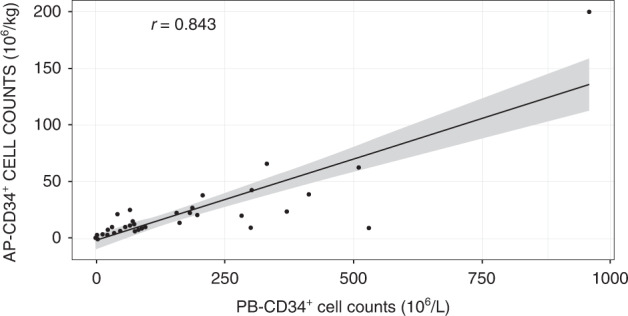
Correlation between PB-CD34^+^ cells and AP-CD34^+^ cell counts the first day of apheresis. AP-CD34+ cluster of differentiation 34^+^ cells on the first day of apheresis, PB-CD34^+^ peripheral blood-cluster of differentiation 34^+^, *r* correlation coefficient.

Based on graphical examination, a linear relationship between the PB-CD34^+^ cell count on the first day of apheresis cells and counts of collected CD34^+^ cells on the first day of apheresis (AP-CD34^+^) was envisaged with a high linear correlation coefficient (*r* = 0.84) between the two variables (Fig. 1). The predictive properties of the linear model were compared with those of log-transformed linear model (correlating log [AP-CD34^+^] with log [PB-CD34^+^]) in the sensitivity analysis. The untransformed linear model showed better predictive performance (by selecting the model with smallest sum of prediction errors absolute values: $\mathop {\sum}\nolimits_i {\left\| {{{{{{\rm{CD34}}}}}}_i - \widehat {{{{{{\rm{CD34}}}}}}_l}_{{{{{\rm{pred}}}}}}} \right.}$) (Table 1) and was thus considered as the base model.

**Table 1:** Comparison of linear and log-transformed models predictive performance.

	Linear model	Log-transformed model
Sum of absolute values of errors (in 10^6^ cells/kg)	398.69	405.76

The base model had the following structure: AP-CD34^+^ = *a* + *b* × (PB-CD34^+^) + *e* where *a, b* are structural model parameters to estimate and *e* is a zero-mean normally distributed residual error term. From the base model, predictive model was enriched by considering some influential covariates and by exploring various types of error structure to describe the residual variability.

### Identification of error structure

The plot of base model residual values, estimated by least-square method, suggested that the standard hypothesis of homogeneous variance did not hold. Therefore, three types of error structure were considered:

- homogeneous variance: *e* = *s* × e where *e* ~ *N* (0,1) and *s* is residual standard error (SE) to estimate.
- proportional error structure: *e* = s × |¦_pred_ | × *e* where *e* ~ *N* (0,1), *σ* is a parameter to estimate and ¦_pred_ is the prediction of AP-CD34^+^ conditional on PB-CD34^+^ (for instance, for the base model *ƒ*_pred = a + b × PB-CD34+_)
- and the other predictors (the additional covariates).
- combined error structure: *e* = *s* × (*k* + |¦_pred_ | ) × *e* where *e* ~ *N* (0,1), *σ* and *κ* are parameters to estimate.

The identification of the error structure was the first step in the model development: the three error structures were assessed using the base model, without any covariate, and the error structure selected was the one associated with the lowest Akaike Information Criterion (AIC).

### Model and covariate selection

Because of the limited sample size, only few covariates were assessed (in addition to PB-CD34^+^ count): age, gender, type of mobilization, tumor type and treatment. The choice of the selected covariates and the final model were driven by AIC minimization, but plausibility and clinical interpretability was also considered.

### Model evaluation

The base model and the final model were evaluated as follows:

- With graphical examination of standard goodness-of-fit plots of the observed values vs the predicted values and of the residuals vs the predicted values.
- The accuracy of the model-based predictions was assessed by comparing the model-based predicted probability of reaching the threshold of 2 × 10^6^ AP-CD34^+^ cells/kg with the observed proportion and its 95% confidence interval (CI); the same comparison was conducted using 5 × 10^6^ AP-CD34^+^ cells/kg threshold.

### Model application

Both base and final models were used to predict and characterize necessary level of circulating PB-CD34^+^ cell counts to reach the threshold of 2 × 10^6^ AP-CD34^+^ cells/kg and 5 × 10^6^ AP-CD34^+^ cells/kg on the first day of apheresis. More precisely the levels of PB-CD34^+^ cell counts that provide a probability of 0.90 to reach 2 × 10^6^ AP-CD34^+^ cells/kg and 5 × 10^6^ AP-CD34^+^ cells/kg were computed with their corresponding 90% CI (90% CI were computed instead of 95% CI because of the limited sample in MOZAIC study). The CIs were computed using Bootstrap approach (by resampling 1000 times the MOZAIC study database).

## Results

The main patient characteristics are described in Table 2. Type of tumor and mobilization were similar between the groups.

**Table 2:** Patient characteristics.

		Plerixafor + G-CSF	G-CSF alone	Total
Age	1 to <6 years	16 (53.3%)	10 (66.7%)	26 (57.8%)
	6 to < 12 years	9 (30.0%)	3 (20.0%)	12 (26.7%)
	≥ 12 years	5 (16.7%)	2 (13.3%)	7 (15.6%)
Sex	Female	11 (36.7%)	8 (53.3%)	19 (42.2%)
	Male	19 (63.3%)	7 (46.7%)	26 (57.8%)
Type of mobilization	G-CSF	23 (76.7%)	10 (66.7%)	33 (73.3%)
	G-CSF/Chemotherapy	7 (23.3%)	5 (33.3%)	12 (26.7%)
Type of tumor	Ewing sarcoma	8 (26.7%)	3 (20.0%)	11 (24.4%)
	Lymphoma	2 (6.7%)	1 (6.7%)	3 (6.7%)
	Neuroblastoma	14 (46.7%)	7 (46.7%)	21 (46.7%)
	Other	6 (20.0%)	4 (26.7%)	10 (22.2%)

*G-CSF* granulocyte colony-stimulating factor.

### Base model

Based on AIC minimization, the combined variability structure was selected. The equation of the estimated base model was AP-CD34^+^ = 1.63 + 1.02 (PB-CD34^+^) + *e* where *e* had a zero-mean normal distribution *N* (0, *s*^2^ × (*k* + prediction)^2^).

The parameter values for intercept, PB-CD34^+^, *k*, and *σ* were 1.63 (SE = 0.72), 0.12 (SE = 0.01), 1.76 and 0.49, respectively.

**Fig. 2:**
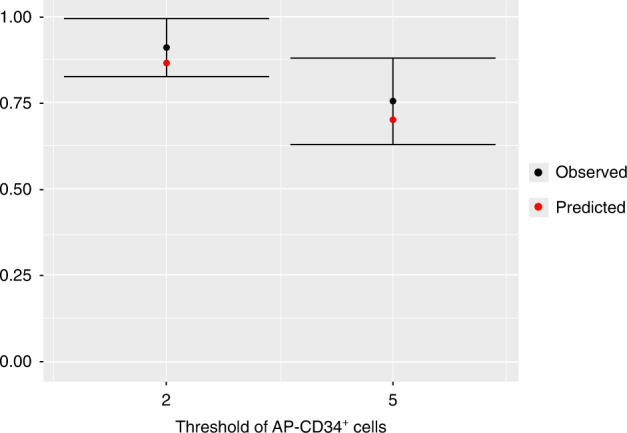
Observed, with 95% CI, and base model predicted proportions of patients achieving 2 × 10^6^ and 5 × 10^6^ AP-CD34^+^ cells/kg. AP-CD34^+^ cluster of differentiation 34^+^ cells on the first day of apheresis, CI confidence interval.

The base model showed a satisfactory goodness-of-fit plot. In the predicted vs the observed scatterplot, the dots were well scattered around the identity line indicating unbiased model predictions. Additionally, the plot of residual values confirmed non-homogeneous variance with greater residual variability for larger predicted values of the AP-CD34^+^ cell counts. In terms of predictive accuracy, the base model was able to properly predict the percentage of patients achieving both 2 × 10^6^ and 5 × 10^6^ AP-CD34^+^ cells/kg (Fig. 2).

**Fig. 3:**
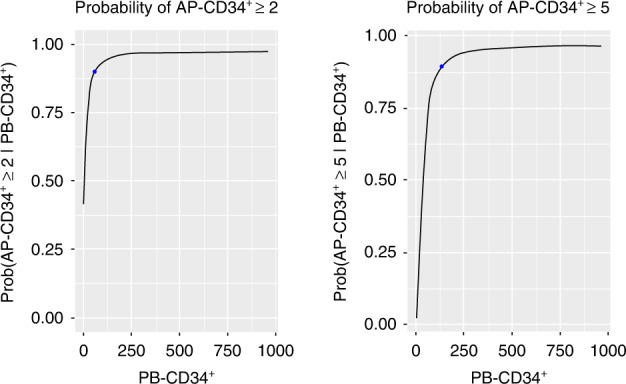
Base model: predicted probability for achieving 2 × 10^6^ and 5 × 10^6^ AP-CD34^+^ cells/kg by PB-CD34^+^ cell counts (in 10^6^ cells/L). AP-CD34^+^ cluster of differentiation 34^+^ cells on the first day of apheresis, PB-CD34^+^ peripheral blood-cluster of differentiation 34.

The base model can be used to characterize the necessary counts of PB-CD34^+^ to achieve thresholds of 2 × 10^6^ and 5 × 10^6^ AP-CD34^+^ cells/kg (Fig. 3).

According to the base model, an estimated PB-CD34^+^ counts of 57.01 (90% CI: 21.76–130.76) and 125.24 (90% CI: 72.09–330.71) × 10^6^/L were necessary to reach thresholds of 2 × 10^6^ and 5 × 10^6^ AP-CD34^+^ cells/kg, respectively, with a probability of 0.90.

#### Final model

Based on AIC minimization, the best model includes the tumor type (neuroblastoma and other) as covariate. The equation of the estimated final model was as follows:

$${{{{{{{\mathrm{Neuroblastoma}}}}}}}}:{{{{{{{\mathrm{AP}}}}}}}} {\mbox{-}} {{{{{{{\mathrm{CD}}}}}}}}34^ + = 3.01 + 0.13 \times \left( {{{{{{{{\mathrm{PB}}}}}}}} {\mbox{-}} {{{{{{{\mathrm{CD}}}}}}}}34^ + } \right) + e$$

$${{{{{{{\mathrm{Other}}}}}}}}\;{{{{{{{\mathrm{tumor}}}}}}}}\;{{{{{{{\mathrm{types}}}}}}}}:{{{{{{{\mathrm{AP}}}}}}}} {\mbox{-}} {{{{{{{\mathrm{CD}}}}}}}}34^ + = 0.01 + 0.13 \times \left( {{{{{{{{\mathrm{PB}}}}}}}} {\mbox{-}} {{{{{{{\mathrm{CD}}}}}}}}34^ + } \right) + e$$

where *e* had a zero-mean normal distribution *N* (0, *s*^2^ × (*k* + prediction)^2^).

The parameter values for intercept-neuroblastoma, intercept-other, PB-CD34^+^, *κ* and *σ* were 3.01 (SE = 1.10), 0.01 (SE = 0.006), 0.13 (SE = 0.01), $\simeq$ 0.00 and 0.54, respectively.

According to the model, the predicted count of AP-CD34^+^ cells was slightly larger for neuroblastoma tumor types than for the other tumor types. It should be noted that the final model was selected considering the type of tumor as an additional covariate (in addition to PB-CD34^+^) based on statistical information criterion (AIC), and that the tumor type was correlated with the age of the patients - the patients with Neuroblastoma tumor type, with mean age of 3.7 years (standard deviation, SD = 2.1 years), being younger than the others with mean age of 8.9 years (SD = 4.8 years). However, the choice of considering tumor type in the final model instead of age was driven by the fact that the fit of the data was improved when tumor type was considered as predictor, as compared to age, which reflected in lower value of the statistical information criterion with tumor type (AIC = 288.6) than with age (AIC = 310.1).

**Fig. 4:**
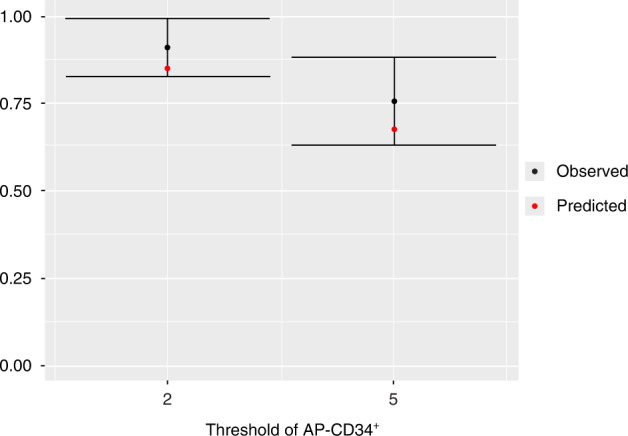
Observed, with 95% CI, and final model predicted proportions of patients achieving 2 × 10^6^ and 5 × 10^6^ AP-CD34^+^ cells/kg. AP-CD34^+^ cluster of differentiation 34^+^ cells on the first day of apheresis, CI confidence interval.

**Fig. 5:**
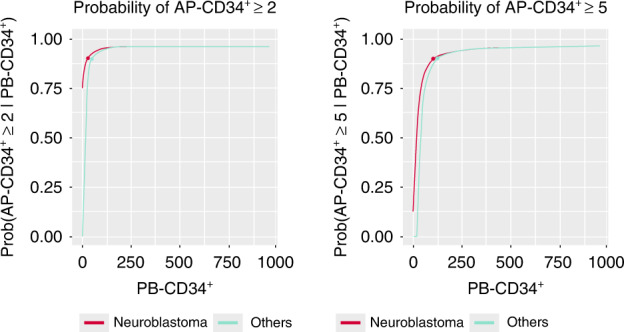
Final model: predicted probability for achieving 2 × 10^6^ and 5× 10^6^ AP-CD34^+^ cells/kg by PB-CD34^+^ cell counts (in 106 cells/L). AP-CD34^+^ cluster of differentiation 34^+^ cells on the first day of apheresis, PB-CD34^+^, peripheral blood-cluster of differentiation 34+.

The final model also showed a good predictive property in terms of goodness-of-fit plot and prediction of the percentages of patients achieving both 2 × 10^6^ and 5 × 10^6^ AP-CD34^+^ cells/kg (Fig. 4). The model predicts that a smaller PB-CD34^+^ cell count was needed to reach 2 × 10^6^ and 5 × 10^6^ AP-CD34^+^ cells/kg with a probability of 0.90 in patients with neuroblastoma tumor type than in those with other tumor types (Fig. 5). According to the final model, in patients with neuroblastoma tumor type, the estimated PB-CD34^+^ counts necessary to reach apheresis thresholds of 2 × 10^6^ and 5 × 10^6^ AP-CD34^+^ cells/kg with a probability of 0.90 were 27.32 (90% CI: 0.16–50.51) and 103.20 (90% CI: 56.15–165.18) × 10^6^/L, respectively. The estimated PB-CD34^+^ counts necessary to reach thresholds of 2 × 10^6^ and 5 × 10^6^ AP-CD34^+^ cells/kg with a probability of 0.90 in patients with other tumor type were 50.51 (90% CI: 29.30–79.12) and 126.39 (90% CI: 77.25–198.28) × 10^6^/L, respectively.

The uncertainty related to these PB-CD34^+^ estimated values with the final model was slightly less in comparison to the base model probably due to a reduced residual variability.

The physiological process of stem cell mobilization via CXCR4 is comparably the same in subjects of all ages, and when adult data on CXCR4 is extrapolated into children it should closely mirror that seen in children [19]. We complemented our analyses with data from the adult NHL and MM patients who participated in the two plerixafor studies [15, 16], focusing on the first day of apheresis similar to the MOZAIC study. The details of the analyses can be found in the Supplementary section.

## Discussion

Repeated attempts at mobilization in poor mobilizers increases resource utilization, morbidity, and patient inconvenience. Higher stem cell doses are associated with faster platelet and neutrophil engraftment [6, 20–24] without increased resource utilization. The use of an algorithm based on PB-CD34^+^ count, the most significant factor correlated to the outcome of mobilization [6], can guide plerixafor use in patients at a higher perceived risk for mobilization failure.

The modeling of the MOZAIC data exhibited a strong and highly predictive linear relationship between PB-CD34^+^ cell count collected on the first day of apheresis and AP-CD34^+^ cells on the same day. The model predicts that there are approximately 13 new collected CD34^+^ cells for 100 new circulating CD34^+^ cells before apheresis.

According to the model, approximately 30 × 10^6^ cells/L and 50 × 10^6^ cells/L of PB-CD34^+^ cells are required to have a probability of ≥0.90 to collect 2 × 10^6^ AP-CD34^+^ cells/kg on the first day of apheresis for the neuroblastoma tumor type and other tumor types, respectively. Additionally, for tumor types other than neuroblastoma, there is no assurance to collect 2 × 10^6^ AP-CD34^+^ cells/kg when the PB-CD34^+^ cell counts are lower than 30 × 10^6^ cells/L. However, there is uncertainty attached to these numbers due to a limited sample size.

Even though our focus was on the pediatric population, similar relationships in adults who received mobilization with either G-CSF alone or G-CSF plus plerixafor was also evaluated. It must be noted that the estimated relationship is quantitatively similar with one obtained by Costa et al. [25], who described the relationship between PB-CD34^+^ cell count on the first day of apheresis and AP-CD34^+^ using mobilization data of 40 adults: “AP-CD34^+^ = –0.39 + 0.13 × PB-CD34^+^.” However, this correlation was observed when 10 patients with PB-CD34^+^ count ≥100 cells/mm^3^ were excluded from the model as their inclusion would have led to a significant distortion in the mathematical correlation between PB-CD34^+^ and AP-CD34^+^ at lower PB-CD34^+^ levels. Furthermore, the slope of 0.13 was very similar to the one estimated from our model. However, an important difference is in the number of apheresis days considered in the two analyses. While the cells were collected over a period of 4 days in Costa et al study, the collection period was limited to a single apheresis day in the MOZAIC study.

A linear relationship between cell counts of PB-CD34^+^ and AP-CD34^+^ collected on the first day of apheresis was confirmed in the two adult studies with a high linear correlation coefficient of ≥75% (Supplementary section*)*. Some differences were noticed concerning the magnitude of the slope and the identification of other predictors (larger sample size allowed a more comprehensive search of covariates in the adult studies) in comparison to the pediatric MOZAIC study. The estimated slope parameter was smaller in the NHL study in comparison to that in the pediatric MOZAIC study. Approximately twice the number of PB-CD34^+^ cells were necessary to collect the same number of AP-CD34^+^ cells, as compared to pediatric MOZAIC study. In the MM study, the model predicted slightly more AP-CD34^+^ cell collection for same amount of PB-CD34^+^ cells when mobilization was conducted with G-CSF plus plerixafor than with G-CSF alone. Also, the model predicted that the count of AP-CD34^+^ collected decreases as age increases for same amount of PB-CD34^+^ cells in this same study. The extrapolation of the predictions to 20 years of age showed that the amount of PB-CD34^+^ cells necessary to reach thresholds of 2 × 10^6^ cells/kg were in the range of those estimated for the pediatric MOZAIC study.

Our study concluded that the count of PB-CD34^+^ cells immediately prior the first day of apheresis was highly predictive of the count of CD34^+^ cells collected on that day in a population of 45 pediatric patients who received either G-CSF alone or G-CSF plus plerixafor. Based on these findings, the best predictive model was linear in PB-CD34^+^ cells count. These findings are consistent with conclusions drawn from the analysis of two studies in adult patients who received either G-CSF alone or G-CSF plus plerixafor. This predictive model can be used to quantify the minimal value of PB-CD34^+^ cells required to collect at least 2 × 10^6^ or 5 × 10^6^ AP-CD34^+^ cells/kg with sufficient assurance (probability = 0.90). However, the strength of our findings is limited because of the small sample size which may induce relatively large predictions intervals. A larger sample size would allow a more comprehensive search of influential factors.

Further validation of our decision-making model with cost analysis can guide the use of plerixafor in patients at a higher perceived risk for mobilization failure along with better utilization of available resources.

## Supplementary information

Supplementary material

Correlation between PB-CD34+ and AP-CD34+ cell counts on the first day of apheresis – non-Hodgkin’s lymphoma study

Final model: Predicted probability for achieving 2 × 106 and 5 × 106 AP-CD34+ cells/kg by PB-CD34+ cell counts (in 106 cells/L)

Correlation between PB-CD34+ and AP-CD34+ cell counts on the first day of apheresis – multiple myeloma study excluding outlier values showing a PB-CD34+ > 500 × 106 cells/L or CD34+ > 100 × 106 cells/

Final model: Predicted probability for achieving 2 × 106 and 5 × 106 CD34+ cells/kg by PB-CD34+ cell counts (in 106 cells/L) - extrapolation for patients aged 20 years

Final model: Predicted probability for achieving 2 × 106 and 5 × 106 AP-CD34+ cells/kg by PB-CD34+ cell counts (in 106 cells/L) – f or patients aged 40 years

Final model: Predicted probability for achieving 2 × 106 and 5 × 106 AP-CD34+ cells/kg by PB-CD34+ cell counts (in 106 cells/L) – for patients aged 60 years

## Data Availability

Qualified researchers may request access to patient-level data and related study documents. Patient-level data will be anonymized, and study documents will be redacted to protect the privacy of trial participants. Further details on Sanofi’s data sharing criteria, eligible studies, and process for requesting access can be found at https://vivli.org/.
