# Supplementary material for: Development and validation of a predictive model to guide the use of plerixafor in pediatric population
Source: Bone Marrow Transplant. 2022 Sep 26;57(12):1827–32. doi: 10.1038/s41409-022-01831-2 (PMC9715428; doi:10.1038/s41409-022-01831-2)
Supplement: Supplementary file 1 — Supplementary material [file 41409_2022_1831_MOESM1_ESM.docx]

**Supplementary material**

The non-Hodgkin’s lymphoma (NHL) study enrolled approximately 300 adults eligible for autologous stem cell transplant who received either granulocyte colony-stimulating factor (G-CSF) alone or G-CSF plus plerixafor in a 1:1 ratio.[^16^](#_ENREF_16) A five-fold increase (range: 0.6–49.8) in peripheral blood-cluster of differentiation 34^+^ (PB-CD34^+^) cell count was previously reported from day 4 to day 5 in the plerixafor-treated patients in comparison to placebo-treated patients (1.4 [range: 0.0–13.0]; *P*<0.001). A graphical examination from the NHL study showed a linear relationship between PB‑CD34^+^ and CD34^+^ harvest collected on the first day of apheresis (AP-CD34^+^) with a high linear correlation (*r* = 0.86; Supplementary Figure S1), similar to the pediatric MOZAIC study. Although this predictive model confirmed the linear relationship between the PB-CD34^+^ and AP-CD34^+^ cell count (Supplementary Figure S2), the estimated slope parameter was smaller in comparison to that in the pediatric MOZAIC study. Approximately, twice the number of PB-CD34^+^ cells were necessary to collect the same number of AP-CD34^+^ cells on the first day of apheresis in the NHL study as compared to pediatric MOZAIC study. Approximately 65 × 10^6^ and 155 × 10^6^ PB-CD34^+^ cells/L provided a probability of 0.90 to reach the thresholds of 2 × 10^6^ and 5 × 10^6^ AP-CD34^+^ cells/kg, respectively.

The multiple myeloma (MM) study enrolled approximately 300 adults eligible for autologous stem cell transplant who received either G-CSF alone or G-CSF plus plerixafor in a 1:1 ratio. The study has previously demonstrated a three-fold increase (from 2.29 to 7.01×10^6^ AP-CD34^+^ cells/kg) in AP-CD34^+^ after inclusion of plerixafor along with G-CSF in the mobilization regimen.[^15^](#_ENREF_15) The linear correlation coefficient (*r* = 0.258; Supplementary Figure S3) in the MM study was observed to be lower than that in the pediatric MOZAIC and NHL studies and appears to be driven by five atypical patients with either much larger PB-CD34^+^ counts (>500 × 10^6^/L) or much larger AP-CD34^+^ counts (>100 × 10^6^/kg) than the other participants of the study. Therefore, the development of the predictive model was conducted after removal of these five outlier patients - by focusing on the set of patients with PB-CD34^+^ counts ≤500 × 10^6^/L and AP-CD34^+^ counts ≤100 × 10^6^/kg. After removal of the outlier patients, the linear *r* was 0.748, similar to the range observed in the pediatric MOZAIC and NHL studies.

Treatment for mobilization (G-CSF plus plerixafor vs G-CSF alone), age, and gender were assessed in addition to PB-CD34^+^. The model predicted slightly more AP-CD34^+^ cell collection for same amount of PB-CD34^+^ cells when mobilization was conducted with G-CSF plus plerixafor than with G-CSF alone. Also, the model predicted that the count of AP-CD34^+^ collected decreases as age increases for same amount of PB-CD34^+^ cells (Supplementary Figures S4, S5 and S6). We observed that approximately 20 × 10^6^ more PB-CD34^+^ cells/kg were required with G-CSF alone, as compared with G-CSF plus plerixafor, to reach thresholds of 2 × 10^6^ and 5 × 10^6^ AP-CD34^+^ cells/kg with a probability of 0.90 (Supplementary Tables S2 and S3). On extrapolation of the predictions to 20 years of age (minimum age in MM study was 28 years), PB-CD34^+^ cells/kg level obtained were in the range of those estimated for the pediatric MOZAIC study (Supplementary Figure S4).

**Supplementary Figure S1: Correlation between PB-CD34^+^ and AP-CD34^+^ cell counts on the first day of apheresis – non-Hodgkin’s lymphoma study**

To develop a predictive model, the following three predictors for CD34^+^ in addition to PB-CD34^+^ were assessed - treatment for mobilization (G-CSF plus plerixafor vs G-CSF alone), age, and gender. Based on the minimization of Akaike Information Criterion (AIC) the final model selected gender and age as additional covariates on top of PB-CD34^+^. However, the estimated coefficient of age was numerically small (with a high *P* > 0.1). Therefore, we decided to ignore the age variable and to consider the model with gender covariate only. The final model can be written as follows:

Female patients: AP-CD34^+^ = 0.07 + 0.07 × (PB-CD34^+^) + *e*

Male patients: AP-CD34^+^ = 0.25 + 0.07 × (PB-CD34^+^) + *e*

where $e$ is zero-mean normally distributed error term, with combined variability structure, similar to the pediatric model. The goodness-of-fit plots showed good predictive performance of the model.
The model predicts a slightly larger number of collected CD34^+^ cells in males than in females for the same amount of PB-CD34^+^ cells. Although statistically significant, this difference is numerically very small and probably not clinically meaningful.

**Supplementary Figure S2: Final model: Predicted probability for achieving 2 × 10^6^ and 5 × 10^6^ AP-CD34^+^ cells/kg by PB-CD34^+^ cell counts (in 10^6^ cells/L).**

******

**Supplementary Figure S3: Correlation between PB-CD34^+^ and AP-CD34^+^ cell counts on the first day of apheresis – multiple myeloma study excluding outlier values showing a PB-CD34^+^ > 500 × 10^6^ cells/L or CD34^+^ > 100 × 10^6^ cells/kg**

Based on AIC minimization, the final model selected arm and age as additional covariates on top of PB-CD34^+^. The final model can be written as follows:

G-CSF: AP-CD34^+^ = 3.11 + 0.05 × (PB-CD34^+^) – 0.04 × Age + *e*

G-CSF + plerixafor: AP-CD34^+^ = 4.25 + 0.05 × (PB-CD34^+^) – 0.04 × Age + *e*

where $e$ is zero-mean normally distributed error term, with combined variability structure, similar to the pediatric model. The goodness-of-fit plots showed good predictive performance of the model.

**Supplementary Figure S4: Final model: Predicted probability for achieving 2 × 10^6^ and 5 × 10^6^ CD34^+^ cells/kg by PB-CD34^+^ cell counts (in 10^6^ cells/L) - extrapolation for patients aged 20 years**

**Supplementary Figure S5: Final model: Predicted probability for achieving 2 × 10^6^ and 5 × 10^6^ AP-CD34^+^ cells/kg by PB-CD34^+^ cell counts (in 10^6^ cells/L)** – **f or patients aged 40 years**

******

**Supplementary Figure S6: Final model: Predicted probability for achieving 2 × 10^6^ and 5 × 10^6^ AP-CD34^+^ cells/kg by PB-CD34^+^ cell counts (in 10^6^ cells/L)** – **for patients aged 60 years**

**Supplementary Table S1: Final model** – **Non-Hodgkin lymphoma study: estimated PB-CD34^+^ cell counts (in 10^6^/L) for achieving 2 × 10^6^ and 5 × 106 AP-CD34^+^ cells/kg and 90% CI**

| **Threshold** | **Male** | **Female** |
| --- | --- | --- |
| 2 × 10^6^ | PB-CD34+ = 65.33  CI: 53.59, 91.30 | PB-CD34+= 67.97  CI: 55.81, 92.70 |
| 5 × 10^6^ | PB-CD34+ = 154.69  CI: 124.69, 231.49 | PB-CD34+= 157.33  CI: 127.18, 232.93 |

Abbreviations: AP-CD34^+^, cluster of differentiation 34^+^ cells on the first day of apheresis; CI, confidence interval; PB-CD34^+^, peripheral blood-cluster of differentiation 34^+^

**Supplementary Table S2. Multiple myeloma study** – **mobilization-CSF plus plerixafor: estimated PB-CD34^+^ cell counts (in 10^6^/L) for achieving 2 × 10^6^ and 5 × 10^6^ AP-CD34^+^ cells/kg and 90% CI**

| **Threshold** | **Age = 20** | **Age = 40** | **Age = 60** |
| --- | --- | --- | --- |
| 2× 10^6^ | PB-CD34^+^ = 45.52  CI: 11.59, 87.46 | PB-CD34^+^ = 60.04  CI: 36.17, 94.27 | PB-CD34^+^ = 74.56  CI: 53.52, 108.39 |
| 5× 10^6^ | PB-CD34^+^ = 168.80  CI: 122.72, 280.03 | PB-CD34^+^ = 183.32  CI: 138.93, 295.94 | PB-CD34^+^ = 197.84  CI: 148.51, 314.07 |

Abbreviations: AP-CD34^+^, cluster of differentiation 34^+^ cells on the first day of apheresis; CI, confidence interval; PB-CD34^+^, peripheral blood-cluster of differentiation 34^+^; G-CSF, granulocyte colony-stimulating factor

**Supplementary Table S3. Multiple myeloma study** – **mobilization-CSF alone: estimated PB-CD34^+^ cell counts (in 10^6^/L) for achieving 2 × 10^6^ and 5 × 10^6^ AP-CD34^+^ cells/kg and 90% CI**

| **Threshold** | **Age = 20** | **Age = 40** | **Age = 60** |
| --- | --- | --- | --- |
| 2× 10^6^ | PB-CD34+ = 66.89  CI: 37.51, 106.24 | PB-CD34+ = 81.41  CI: 59.95, 116.47 | PB-CD34+ = 95.92  CI: 71.73, 132.58 |
| 5× 10^6^ | PB-CD34+ = 190.16  CI: 140.36, 307.30 | PB-CD34+ = 204.68  CI: 151.60, 321.86 | PB-CD34+ = 219.20  CI: 160.92, 340.71 |

Abbreviations: AP-CD34^+^, cluster of differentiation 34^+^ cells on the first day of apheresis; CI, confidence interval; PB-CD34^+^, peripheral blood-cluster of differentiation 34^+^; G-CSF, granulocyte colony-stimulating factor
