# Supplementary material for: Development and validation of a predictive model to guide the use of plerixafor in pediatric population
Source: Bone Marrow Transplant. 2022 Sep 26;57(12):1827–32. doi: 10.1038/s41409-022-01831-2 (PMC9715428; doi:10.1038/s41409-022-01831-2)
Supplement: Supplementary file 2 — Correlation between PB-CD34+ and AP-CD34+ cell counts on the first day of apheresis – non-Hodgkin’s lymphoma study [file 41409_2022_1831_MOESM2_ESM.pdf]

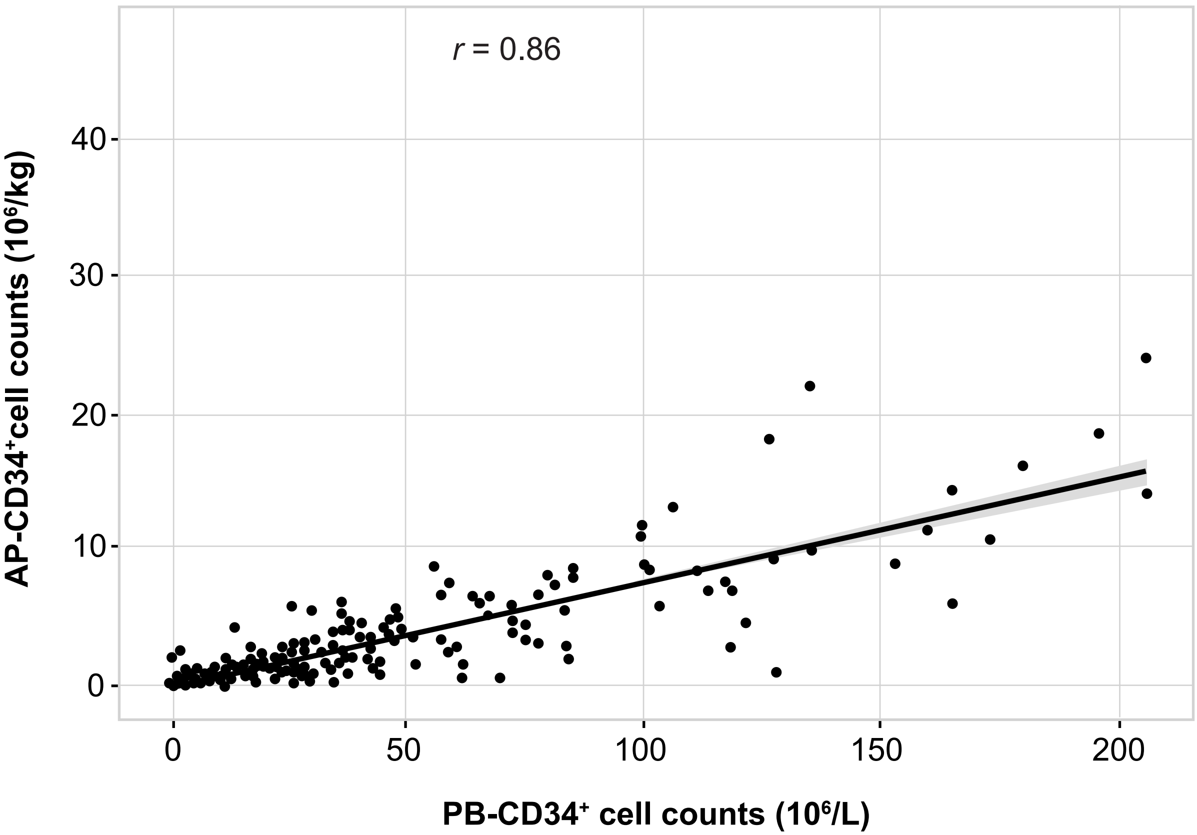

AP-CD34$^+$, cluster of differentiation 34$^+$ cells on the first day of apheresis; PB-CD34$^+$, peripheral blood-cluster of differentiation 34$^+$; *r*, correlation coefficient.
