# Supplementary material for: Development and validation of a predictive model to guide the use of plerixafor in pediatric population
Source: Bone Marrow Transplant. 2022 Sep 26;57(12):1827–32. doi: 10.1038/s41409-022-01831-2 (PMC9715428; doi:10.1038/s41409-022-01831-2)
Supplement: Supplementary file 4 — Correlation between PB-CD34+ and AP-CD34+ cell counts on the first day of apheresis – multiple myeloma study excluding outlier values showing a PB-CD34+ > 500 × 106 cells/L or CD34+ > 100 × 106 cells/ [file 41409_2022_1831_MOESM4_ESM.pdf]

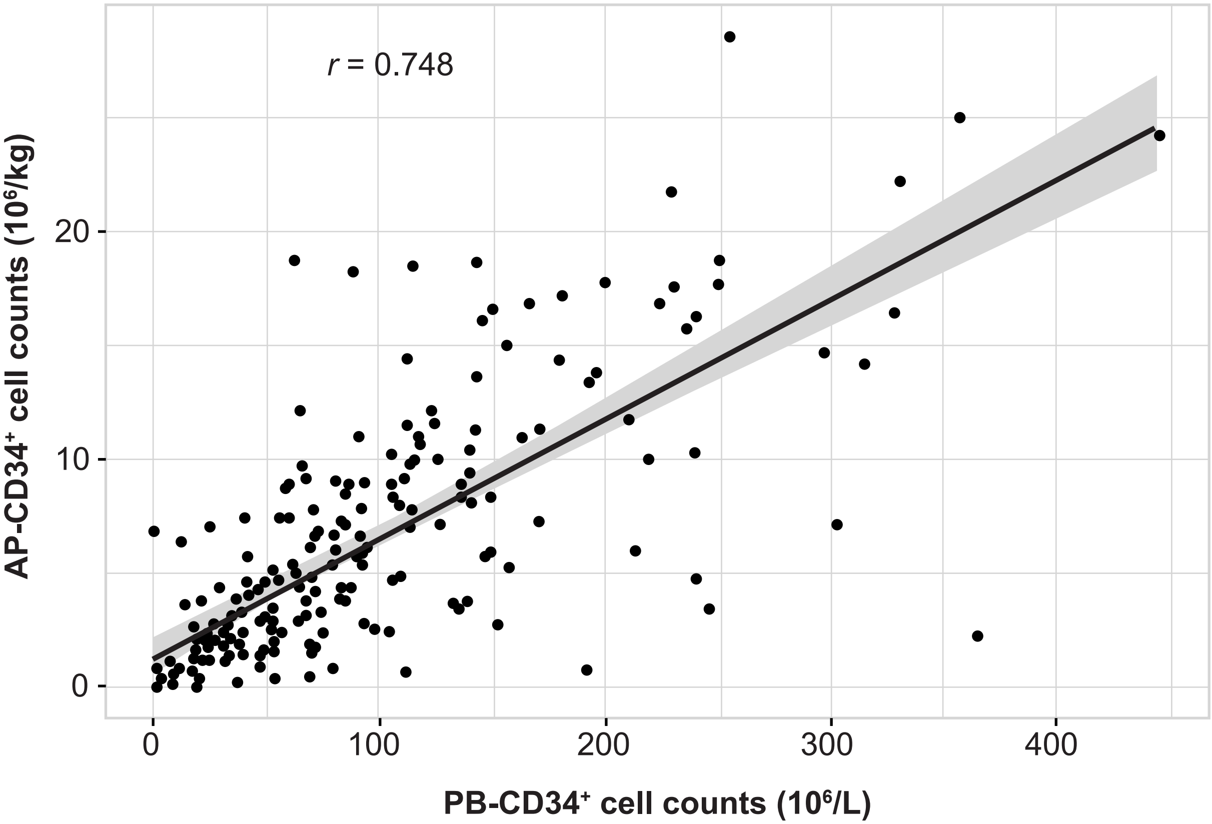

AP-CD34⁺, cluster of differentiation 34⁺ cells on the first day of apheresis; PB-CD34⁺, peripheral blood-cluster of differentiation 34⁺; *r*, correlation coefficient.
