# Supplementary material for: Development and validation of a predictive model to guide the use of plerixafor in pediatric population
Source: Bone Marrow Transplant. 2022 Sep 26;57(12):1827–32. doi: 10.1038/s41409-022-01831-2 (PMC9715428; doi:10.1038/s41409-022-01831-2)
Supplement: Supplementary file 6 — Final model: Predicted probability for achieving 2 × 10^6^ and 5 × 10^6^ AP-CD34+ cells/kg by PB-CD34+ cell counts (in 106 cells/L) – f or patients aged 40 years [file 41409_2022_1831_MOESM6_ESM.pdf]

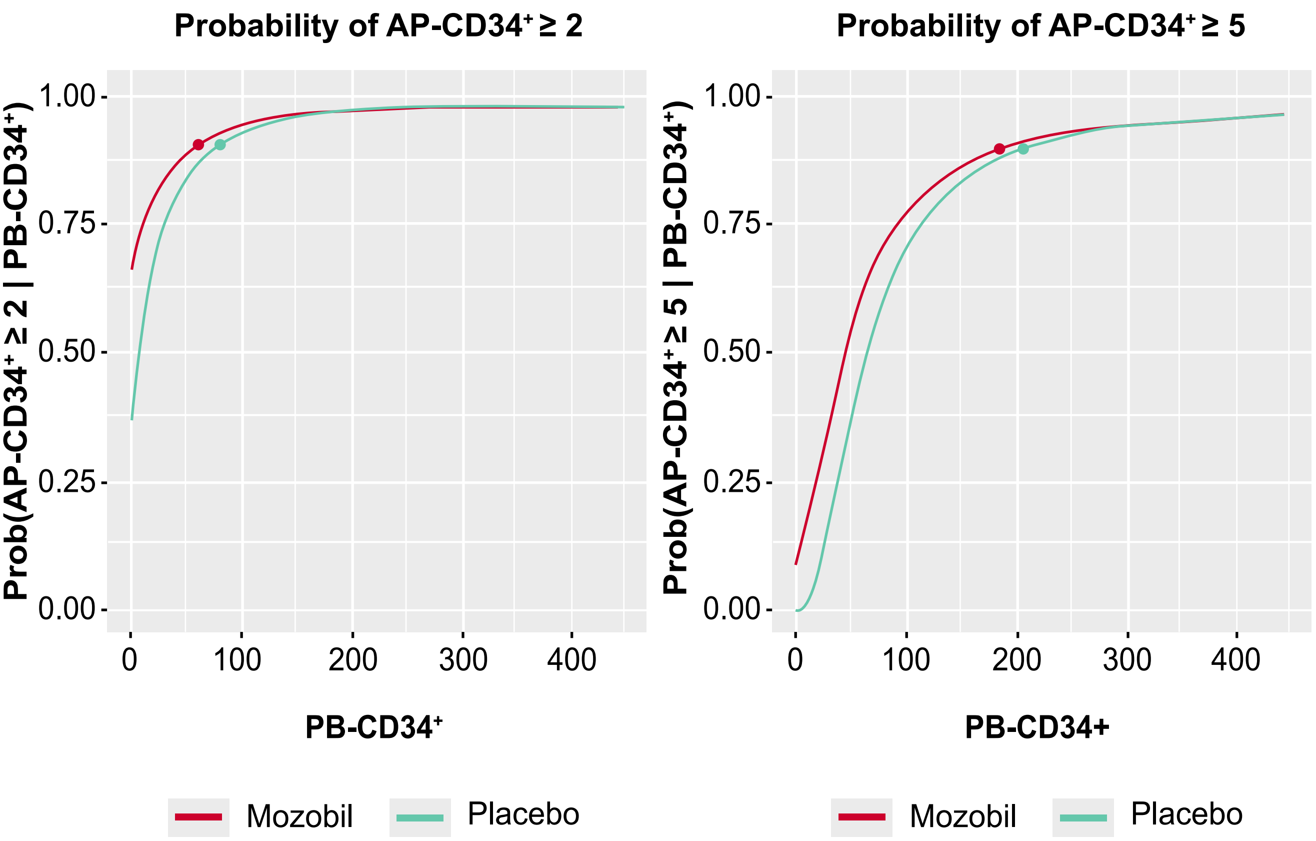

AP-CD34⁺, cluster of differentiation 34⁺ cells on the first day of apheresis; PB-CD34⁺, peripheral blood-cluster of differentiation 34⁺
